# Relationship of mTORC1 and ferroptosis in tumors

**DOI:** 10.1007/s12672-024-00954-w

**Published:** 2024-04-07

**Authors:** Huilin Liao, Yueqing Wang, Lili Zou, Yanmei Fan, Xinyue Wang, Xiancong Tu, Qiaobai Zhu, Jun Wang, Xiaowen Liu, Chuanjiang Dong

**Affiliations:** 1 Hubei Key Laboratory of Tumor Microenvironment and Immunotherapy, College of Basic Medical Science, China Three Gorges University, Yichang, Hubei China 443002; 2 Department of Urology, The First Dongguan Affiliated Hospital of Guangdong Medical University, Dongguan, Guangdong China 523000; 3 The Institute of Infection and Inflammation, College of Basic Medical Sciences, China Three Gorges University, Yichang, Hubei China 443002; 4 The People’s Hospital of China Three Gorges University and The First People’s Hospital of Yichang, Yichang, Hubei China 443002

**Keywords:** Ferroptosis, mTOR, AMPK, Autophagy, GPX4, Tumors

## Abstract

Ferroptosis is a novel form of programmed death, dependent on iron ions and oxidative stress, with a predominant intracellular form of lipid peroxidation. In recent years, ferroptosis has gained more and more interest of people in the treatment mechanism of targeted tumors. mTOR, always overexpressed in the tumor, and controlling cell growth and metabolic activities, has an important role in both autophagy and ferroptosis. Interestingly, the selective types of autophay plays an important role in promoting ferroptosis, which is related to mTOR and some metabolic pathways (especially in iron and amino acids). In this paper, we list the main mechanisms linking ferroptosis with mTOR signaling pathway and further summarize the current compounds targeting ferroptosis in these ways. There are growing experimental evidences that targeting mTOR and ferroptosis may have effective impact in many tumors, and understanding the mechanisms linking mTOR to ferroptosis could provide a potential therapeutic approach for tumor treatment.

## Introduction

mTOR, a mammalian target protein of rapamycin, is a direct target protein of the rapamycin-FKBP12 (12 kDa FK506 binding protein) complex, first reported in 1995 by RT Abraham's laboratory [1]. It is a central regulator of cell growth and apoptosis, a typically conserved serine/threonine kinase, of which, mTOR is part of the phosphatidylinositol 3-kinase-related kinase (PIKK) protein family, mainly regulates the control of cellular metabolism, stress and other environmental influences such as protein [2].

Unlike other apoptosis, necrosis or autophagy, ferroptosis is a novel form of iron-dependent programmed death [3]. The unique form of ferroptosis is reflected in the accumulation of iron ions, the accumulation of lipid reactive oxygen species (ROS), and lipid peroxidation. The involvement of mTOR in cellular autophagy has been demonstrated [4]. Besides, there is a close relationship between autophagy and ferroptosis [5]. mTOR inhibition can lead to autophagy or ferroptosis through different downstream pathways, and increased intracellular lipid peroxidation due to autophagy can also lead to ferroptosis.

In this review, we systematically introduce the basic relationship of mTOR and ferroptosis, hopefully offering some hope in the clinical treatment of tumors [6].

## Core mechanisms of mTOR

There are two main multi-complex proteins of mTOR, mTORC1 and mTORC2. The main target of rapamycin is mTORC1, which plays a major role in cellular transduction. mTORC1 is mainly composed of mTOR, raptor (regulatory protein associated with mTOR), promotes substrate recruitment) and mLST8 (mammalian lethal Sec13 protein 8, also known as GßL) [7].

mTOR can coordinate protein synthesis, improve intracellular energy status, participate in lipid metabolism to control cell growth and metabolic activities through various metabolic pathways, including AMPK, PI3K/AKT, TSC1, etc. [8]. For example, The activation of mTOR can phosphorylate eIF4E-binding proteins (4E-BPs) to promote eIF4F formation,which induces the initiation of protein synthesis [9, 10]. In addition, in the state of cell energy stress, AMPK is stimulated resulting in inhibition of mTOR by phosphorylating directly and phosphorylating TSC2 at Ser1387 indirectly [11, 12] More importantly, over-expression of mTOR is often found in tumors, such as pituitary adenomas and penile squamous cell carcinoma [13, 14].

The mTOR modulators have been used to treating diverse diseases, including leukemia etc [15, 16]. HGF stimulates mTOR resulting in inhibition of pyroptosis in septic [17]There is also a correlation between mTOR modulators and ferroptosis. AZD8055, a modulator of novel ATP-competitive inhibitor of mTOR, which induces ferroptosis via p70S6K and 4E-BP1 pathway [18]. Besides, mTOR can up-regulate the generation of ISC through ISCU, which results in decreasing the expression of iron transport-associated genes and inhibits ferroptosis [19]. In conclusion, the relationship between mTOR and ferroptosis cannot be ignored.

## Core mechanisms of Ferroptosis

Ferroptosis, a clinically promising cancer cell endpoint for treatment, has great expectations in the treatment of tumors. Aberrant ferroptosis in diverse cancer types and tissues has been summarized in other reports. Gao W et al. have summarized the association between immunity, ferroptosis and autophagy in tumor therapy. There also has been reported that targeting ferroptosis can treat liver cancer [20–22].

The main factors responsible for ferroptosis in cells are free iron ions and intracellular ROS. Iron ions play an irreplaceable role in the development of ferroptosis in cells by first binding to transferrin (TF), and then binding to transferrin receptors on the cell membrane to mediate iron ions into the cell [23]. Fe(III) subsequently can be converted to Fe(II) in endosomes by STEAP3 [24]. Ferritin (FT), consisted of ferritin heavy chain (FTH) and ferritin light chain (FTL), has ferric oxidase activity that converts Fe(II) to Fe(III), which can store excess intracellular iron ions to regulate iron homeostasis strictly [25]. Nuclear receptor coactivator 4 (NCOA4) is a selective receptor for lysosomal autophagic conversion of ferritin. Once intracellular irons exhausted, ferritin undergoes autophagic degradation via NCOA4 thereby releasing Fe(II) to increased free iron levels [24]. This process is ferritinophagy, which promotes ferroptosis via the Fenton reaction, resulting in ROS accumulation and lipid peroxidation.

Another very critical factor for ferroptosis is ROS, and one of the sources of intracellular ROS production is indirectly generated from free iron ions by the Fenton reaction that Fe(II) remove peroxide bonds of H_2_O_2_ [26, 27]. When the intracellular ROS level exceeds the cell's own regulation, ROS tend to attack polyunsaturated fatty acids (PUFA) and phosphatidylethanolamine (PE) on cell membranes or organelles, thus causing lipid peroxidation, resulting in cellular damage and ferroptosis [28]. Based on it, more and more researchers are trying to use drugs to increase the level of ROS to target ferroptosis to treat tumors. In ovarian cancer, Ying Liu et al. used Agrimonolide to increase ROS levels to stop ovarian cancer cells migration and progression [29]. In addition, Trabectedin drug can induce ferroptosis in non-small cell lung cancer (NSCLC) by up-regulating the level of ROS [30].

Correspondingly, the elimination of lipid peroxidation plays an important role in inhibition of ferroptosis. GPX4, which reduces peroxidized lipids, repairs oxidatively damaged lipids, and glutathione (GSH) is involved in its reduction process as a cofactor of GPX4 from GSH to GSSH [31]. It has been demonstrated that the concentration of glutathione depends on the concentration of cysteine, while the cellular uptake of cysteine depends on the cysteine/glutamate transporter protein system (System Xc^−^), which consists of SLC3A2 and SLC7A11 [32]. When cysteine is inhibited from entering the cell, GSH synthesis is reduced thereby leading to inactivation of GPX4 and resulting in lethal levels of ROS, which ultimately induces the onset of ferroptosis [33].

## Mechanism linking mTOR to ferroptosis

### Inhibition of PI3K/AKT/mTOR signaling pathway promotes ferroptosis

The PI3K/AKT/mTOR signaling pathway is activated or inhibited in a variety of tumors. The PI3K/AKT/mTOR pathway has been reported to be involved in the regulation of intracellular oxidative stress, maintaining redox homeostasis [34]. As one of the most mutated pathways among cancer cells, the PI3K/AKT/mTOR signaling pathway often results more resistant to ferroptosis in cells [7, 35, 36].

Firstly, PI3K/AKT/mTOR can inhibit ferroptosis through Sterol regulatory element binding protein 1 (SREBP1)/Stearoyl-CoA desaturase-1 (SCD1)-mediated lipogenesis [37]. SREBP1, a major regulator of cellular metabolism (glucose or lipids, etc.), can be activated by specific protein hydrolysis to sense intracellular metabolic signals [38]. SCD1 is an enzyme that converts saturated fatty acids to monounsaturated fatty acids (MUFAs), which can reduce cellular susceptibility to ferroptosis in lung and gastric cancer cells [39–41]. Knockdown of SREBP1 can downregulate SCD1 expression at the mRNA and protein levels, thus reducing MUFAs to promote cellular ferroptosis [41]. In contrast, signal phospholipid 5A (Semaphorin 5A), enhances SREBP1/SCD1 signaling by activating the PI3K/AKT/mTOR signaling axis, leading to activation of fibroblasts thereby inhibiting ferroptosis of fibroblasts [42].

Besides, quinazolinyl arylurea derivatives, synthesized based on structural modifications of sorafenib, promoted ferroptosis through PI3K/AKT/mTOR/ULK1 pathway [43].

To sum up, inhibition of the PI3K/AKT/mTOR signaling pathway to induce ferroptosis provides a potential therapeutic approach for the treatment of tumors.

### AMPK/mTOR signaling pathway mediates ferroptosis

Cellular redox levels have a profound effect on autophagy. Besides, lipid peroxidation can induce autophagosome formation. Correspondingly, autophagy also be induced by ferroptosis through lipid peroxidation and iron ion accumulation [44, 45]. Ferritinophagy, is the one of ways that autophagy regulating ferroptosis. Moreover, damaged mitochondrias lead to elevated ROS, which results cellular autophagy and inducing ferritinophagy thereby causing ferroptosis in pancreatic cancer cells [46]. AMPK has close relationship with energy level in cells, so the AMPK/mTOR pathway plays a huge role in the occurrence of ferroptosis and autophagy in cells. The adenosine-monophosphate-activated protein kinase (AMPK) regulates cellular autophagy through downstream signaling molecules of mTOR, such as unc-51-like kinase (ULK1), P70S6K, to regulate ferroptosis [47, 48].

ULK1 is a direct target of many kinases and functions in several stages of autophagy to initiate autophagosome binding [49]. AMPK has been reported to phosphorylate ULK1 Ser317 and Ser777 to induce cellular autophagy [50, 51]. Bisphenol A (BPA) may increase phosphorylation of AMPK and ULK1 to induce autophagy and ferroptosis through the AMPK/mTOR/ULK1 pathway in ovarian granulosa cells (GC) and renal tubular epithelial cells [47, 52, 53]. On the other hand, BPA can lead to NCOA4-mediated degradation of FTH, resulting in an increase of intracellular iron ion and thus ferroptosis [53]. Copper sulfate (CuSO_4_) has also been reported to induce autophagy and ferroptosis through the same pathway with BPA [51].

P70S6K is a serine/threonine kinase that regulates ribosomal protein S6 translation and thus controls cell cycle and migration ability [54, 55]. It was demonstrated that phosphorylated AMPK reduced the phosphorylation of P70S6K, a signaling molecule downstream of mTOR, to promote autophagy with LC3I changed to LC3-II [48], and to downregulated SLC7A11 expression to induce ferroptosis [56].

In addition, there are other compounds that are closely associated with AMPK or mTOR. SIRT3, a NAD-dependent mitochondrial protein deacetylase, regulated ROS production [57, 58]. Reduction of protein level of SIRT3 inhibits the activation of AMPK/mTOR signaling pathway and thus inhibits cells from undergoing autophagy-dependent ferroptosis [59]. Amenflavone (AF) was found to promote ferroptosis with the signaling pathway AMPK/mTOR in glioma cells and endometrial cancer cells [60, 61]. NRF2 is a stress-induced transcription factor that controls numbers of enzymes involved in GSH synthesis [62]. Silver-coated (ZVI-NP) individual nanodrugs can disrupt the AMPK/mTOR signaling pathway to enhance the degradation of NRF2, thus inducing intracellular mitochondrial dysfunction to ferroptosis [63].

### Combined targeting of GPX4 and mTOR leads to ferroptosis

GPX4, as one important regulator of ferroptosis, cannot be activated without selenocysteine [64], which can induce cellular autophagy through AMPK/mTOR [65]. It was found that inhibiting mTOR signaling pathway upregulated GPX4 and SOD expression to inhibit ferroptosis in cells [64]. Besides, it has been proposed that inhibition of mTORC1 can downregulate GPX4 expression [66]. Torin1 and Rapamycin, both could inhibit the phosphorylation of the mTOR signal pathway, were found to downregulate GPX4 expression [66–68]. A recent study also found that silencing of GPX4 and inhibition of mTOR increased the sensitivity of ferroptosis [69]. BECN1, involved in autophagy, can be phosphorylated by AMPK at Ser90 and Ser93 resulting in inhibition of system Xc, which reduces production of GSH and inhibition of GPX4 [70].

In conclusion, GPX4 expression and mTOR activation were positively correlated. Inhibition of mTOR and downregulation of GPX4 protein synergistically can be a potential therapeutic mechanism for anti-tumor.

### Other factors related mTOR and ferroptosis

Epidermal growth factor receptor (EGFR), a receptor tyrosine kinase, is closely related to cellular autophagy [71]. Studies showed that GALNT14 could downregulate glycosylation of EGFR to inhibit mTOR activity, causing autophagy and ferroptosis [72]. It was also reported that EGFR inhibition could enhance cellular sensitivity to ferroptosis by upregulating LC3B-II to induce autophagy [73]. Therefore, there is a correlation between EGFR and mTOR.

Besides, there is a correlation between transcription factors (TFEB) and mTOR. Activation of mTORC1 can lead to TFEB phosphorylation to inhibit transcription of target genes [74]. Studies showed that polystyrene nanoparticles (CPS) inactivated mTORC1 to induce TFEB dephosphorylation, causing upregulation of SOD expression and ultimately inhibiting ferroptosis [75].

There is a tight relationship between p53 and mTOR [76]. p53-mediated cell death can be blocked by the AKT/mTOR pathway [77]. What’s more, p53 has been shown to be closely associated with cellular activities, including cell cycle arrest, nucleotide metabolism, apoptosis and ferroptosis [78–80].

It has been reported that mTORC1 can facilitate P62 to enable KEAP1 binding leading to NRF2 accumulation thereby directing the antioxidant pathway [81]. Inhibition of mTORC1 makes cells more sensitive to ferroptosis as an independent mechanism relative to the NRF2-mediated signaling pathway.

**Fig. 1:**
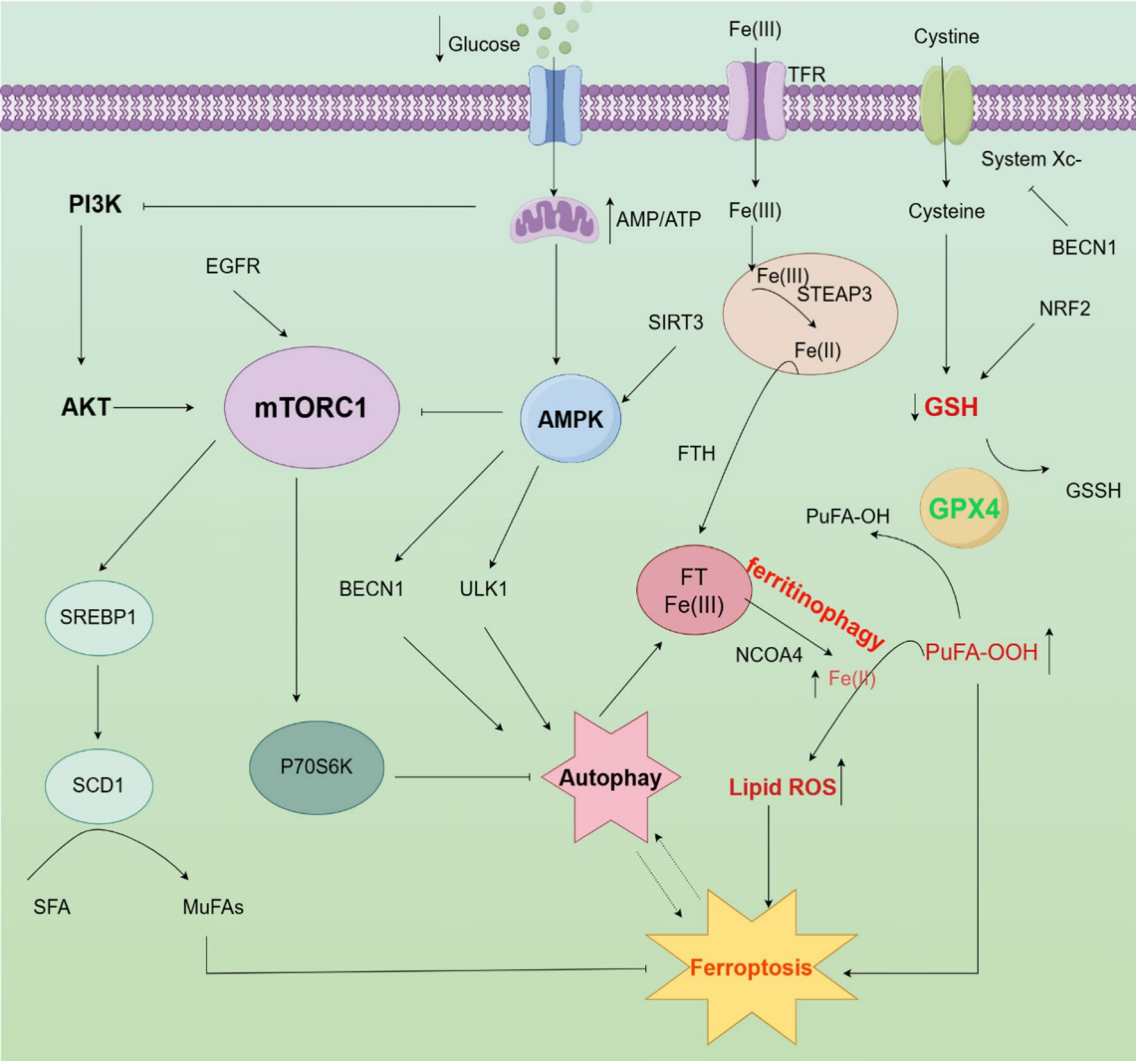
The mechanism of mTOR associated with ferroptosis. Fe(III) is taken up by TFR, converted into Fe(II) by STEAP3 in the endosome, and finally stored in FT. Under the condition of low energy, AMPK is activated, which activates BECN1 and ULK1 and inhibits mTORC1 to promote cellular autophagy. BECN1 activation inhibits System Xc- and reduces GSH synthesis, which leads to increased PuFA-OOH and promotes ferroptosis. Upon cellular autophagy as well as intracellular Fe exhaustion, lysosomes bind to FT releasing large amounts of Fe(II) thereby resulting in the Fenton reaction leading to an increase in Lipid ROS, leading to ferroptosis. PI3K/AKT activation of mTORC1, mTOR downstream SREBP1 and SCD1 activation converts SFA into MuFAs, and P70S6K activation ultimately inhibits ferroptosis and autophagy. TFR, transferrin receptor; Lipid ROS, lipid reactive oxygen species; PUFA, poly-unsaturated fatty acid; MUFA, monounsaturated fatty acid; NRF2, nuclear factor erythroid 2-related factor 2; NOCA4, nuclear receptor coactivator 4; GPX4, glutathione peroxidase 4; GSH, glutathione; GSSH, oxidized glutathione; FTH, ferritin heavy chain; SREBP, sterol responsive element binding protein; ULK1, unc-51 like autophagy activating kinase 1; AMPK, AMP-activated protein kinase; BECN1, beclin 1; mTORC1, mechanistic target of rapamycin complex 1; (By Figdraw)

In summary, mTOR is regulated by many factors and mTOR interacts with them to regulate ferroptosis (Fig. 1).

## mTOR-related drugs induced ferroptosis in various tumors

As mentioned earlier, ferroptosis has close relationship with mTOR. Thus, using mTOR-related drugs is possible to promote the occurrence of ferroptosis in the treatment of various tumors (Table 1).

**Table 1:** Drugs/proteins associated with mechanism of ferroptosis and mTOR in tumors

Drugs/proteins	Cell lines /tissues	Target	Effect	References
CCI-779	BT474	↓mTOR	Inducing ferroptosis	[98]
Semaphorin 5A	Synovial Fibroblasts (SFs)	↓PI3K/AKT/mTOR	Inhibiting ferroptosis	[42]
Melatonin (MT)	Osteoblasts and Bone Marrow Mesenchymal Stem Cells (BMSCs)	↓PI3K/AKT/mTOR	Inhibiting ferroptosis	[109]
Compound 7j	T24	↓Sxc/GPX4/ROS↓PI3K/Akt/mTOR/ULK1	Inducing ferroptosis/autophagy/apoptosis	[43]
Tocopherol	BMSC	PI3K/AKT/mTOR	Inhibiting ferroptosis	[110]
DHA	HL60/ THP-1	↑AMPK/mTOR/p70S6k	Promoting autophagy/ferroptosis	[48]
IMCA	DLD-1/HCT-116	↓SLC7A11↑AMPK/mTOR/p70S6k	Inducing ferroptosis	[56]
Bisphenol A (BPA)	TCMK-1	↑AMPK/mTOR/ULK1	Promoting autophagy/ferroptosis	[53]
CuSO4	GC-1 spg cells	↑AMPK/mTOR/ULK1	Promoting autophagy/ferroptosis	[51]
Sirtuin 3 (SIRT3)	HTR8	↑AMPK/mTOR↓GPX4	Promoting autophagy/ferroptosis	[59]
AF	U251/U373 glioma; Endometrial Carcinoma Cells	↑AMPK/mTOR	Promoting autophagy/ferroptosis	[60, 61]
Zero-Valent-Iron Nanoparticle (ZVI-NP)	MRC-5 and IMR-90	↑AMPK/mTOR	Inducing ferroptosis	[63]
GNA	B16F10	↓SLC7A11↑AMPK/mTOR	Promoting autophagy/ferroptosis	[111]
Everolimus	HEK-293	↓mTOR/4EBP1	Inducing ferroptosis	[100]
Bupivacaine	T24/5637	↓PI3K/Akt	Inducing apoptosis/ferroptosis	[92]
GALNT14	SKOV3/OVCAR-3	↓EGFR/mTOR	Inducing apoptosis/ferroptosis	[72]
Lapatinib/PAB@Ferritin	MDA-MB-231	↓YAP/mTOR	Promoting autophagy/ferroptosis	[73]
Carboxyl-modified Polystyrene nanoparticles (CPS)	RAW264.7	↓mTORC1	Inhibiting ferroptosis	[75]
Aspirin	DLD-1/HCT 116	↓PI3K/AKT/mTOR/SREBP/SCD1	Inducing ferroptosis	[84]
RSL3	PANC/MIAPaCa	↓mTOR	Promoting autophagy/ferroptosis	[66]
Levobupivacaine	MCF-7 and MDA-MB231	↓PI3K/AKT/mTOR/Bax	Inducing ferroptosis	[94]
Small-Molecule Inhibitor V9302	Triple-negative Breast Cancer; Breast Cancer Stem-Like Cell	↓mTOR	Inducing ferroptosis	[97]

### Colorectal cancer

Colorectal cancer (CRC) is one of the leading causes of death among cancers worldwide [82].

IMCA, a benzopyran derivative, was discovered to control apoptosis through ferroptosis [83]. On the one hand, it has been found that IMCA can reduce GPX4 synthesis by downregulating SLC7A11. On the other hand, IMCA phosphorylated AMPK to inhibit mTOR/P70S6K activity thereby inducing ferroptosis in CRC [56].

What’s more, inhibition of mTOR signaling by Aspirin monounsaturated fatty acid production to promote ferroptosis through PI3K/AKT/mTOR/SREBP/SCD1 signaling pathway in CRC [84]. Thus, ferroptosis could be a new hope for clinical treatment of colorectal cancer.

### Pancreatic cancer

Pancreatic cancer (PC) is one of the most aggressive and lethal malignancies with extremely poor prognosis, the most common pathological type of PC is pancreatic ductal adenocarcinoma (PDAC) [85]. studies have demonstrated that PDAC is mainly dependent on the uptake of Cysteine, closely linked to GPX4 [86]. Besides, the development of PDAC can be inhibited by using cyst(e)inase, which has been reported to eliminate circulating cysteine and cysteine [86, 87].

A recent study, attempted to use a combination of starvation and ferroptosis for treatment, found that pancreatic cancer cells with different levels of starvation have different sensitivity to ferroptosis and that mTORC2-mediated signaling pathways are involved in this mechanism [88]. Precise molecular pathways induced by ferroptosis may provide new insights into the treatment of PC.

### Bladder cancer

Bladder cancer is one of the malignant tumors in the urological system, with poor prognosis and high risk of recurrence after surgery [89]. In recent years, it has been found that type III ferroptosis inducer FIN56 and mTOR inhibitor-Torin2 can induce autophagic ferroptosis by decreasing GPX4 and inhibiting mTOR leading to decreased phosphorylation of ULK1 in bladder cancer [90]. Baicalein has been reported to induce cellular ferroptosis through degrading FTH1 to induce the accumulation of ROS and iron ions in cancer cells [91]. Bupivacaine has been reported to induce ferroptosis by inhibiting the PI3K/AKT pathway, accompanied by decreasing mTOR phosphorylation in bladder cancer cells [92].

In conclusion, drugs related to ferroptosis and mTOR have provided hope in the treatment of bladder cancer.

### Breast cancer

Breast cancer is the most lethal tumor in women [93]. Studies have showed that Levobupivacaine inhibited the proliferation and induce apoptosis of breast cancer cells by inhibiting the PI3K/AKT/mTOR signaling pathway [94]. Besides, the combination of Selamethicin and Lapatinib can synergistically downregulate iron transport protein and upregulate TF to increase the accumulation of intracellular ROS and iron ions to induce ferroptosis in breast cancer cells [95].

Triple negative breast cancer (TNBC) is one of the more aggressive types of breast cancer [96]. A recent study used the small molecule inhibitor V9302 to inhibit glutamine uptake and inhibit mTOR, thus inducing ferroptosis in TNBC cells [97]. In addition, ferritin nanoparticles (L/P@Ferritin) could inhibit the YAP/mTOR pathway to reduce EGFR activity and regulate ferroptosis in TNBC cells [73].

In conclusion, the above new anticancer drugs offer hope for the treatment of clinical breast cancer.

### Prostate cancer

Prostate cancer, the more common tumors in men, is one of the mot malignant tumors in urological system [85]. A recent work found that it is possible to virtually eliminate tumors by combined treatment with mTOR inhibition and ferroptosis, through inhibiting PI3K/AKT/mTOR to downregulate SCD1 and SREBP1 for inducing ferroptosis [98]. Ferroptosis inducer RSL3 or Erastin, combined with anti-androgens together, showed good efficacy to inhibit prostate cancer [99].

Thus, the molecular mechanism regarding mTOR and ferroptosis could provide a new target for prostate cancer treatment.

### Other tumors

There are some other tumors related ferroptosis and mTOR. As mentioned before, GALNT14 could inhibit mTOR activity to cause ferroptosis in ovarian cancer [72]. Besides, the combination of Everolimus and RSL3/Erastin can induce ferroptosis in renal cell carcinoma through the mTOR/4EBP mechanism [100]. In endometrial cancer, Brass has been reported to activate the ROS/AMPK/mTOR pathway to inhibit the viability and invasion of cancer cells and promote their ferroptosis. In glioma cells, AF can also play a role in inducing ferroptosis by the AMPK/ mTOR/ p70S6K signaling pathway [60, 61].

## Conclusion and outlook

In glioblastoma, activation of the mTOR pathway contributes to cancer cell growth. [101]. In addition, from clinical data, compared with normal tissues, the level of phosphorylated mTOR is higher in penile squamous cell carcinoma [13]. Besides, the application of combined targeting of mTOR and ferroptosis have a good prospect in tumors, such as ovarian cancer, triple-negative breast cancer, bladder cancer, etc.

In the way of drugs targeting mTOR, mTORC1 is more sensitive to rapamycin as the raptor [102]. As for mTOR pathway is very complex and different tumor cells have different sensitivity to rapamycin, rapamycin alone has a low success rate in inhibiting cancer cell proliferation [103]. In addition, it has been reported that the mTORC1 inhibitor-adriamycin inhibits cell translation and affects the phosphorylation of eIF2α to promote cell migration in MCF10A cancer cells [104]. On the contrary, researchers also found that inhibition of mTOR suppressed the production of ferroptosis in cells [105]. Under conditions of mTOR inhibition, using Class I FIN to inhibit System Xc- to prevent entry of cystine, which results in the more synthesis of GSH from mTOR downstream amino acids, thus inhibiting ferroptosis [105].

In addition, ROS levels in tumors is complex, and some cancer cells also have higher levels of ROS to promote tumor development [106]. For example, triple-negative breast cancer exhibits dependency on ROS level to survival [107]. In sum, it is not yet clear how to target mTOR for cancer therapy by regulating ROS levels. Many researchers are trying to find other inhibitors to inhibit tumor growth in combination with rapamycin [108]. Thus, we summarize the related mechanisms of mTOR and ferroptosis, including PI3K/AKT/mTOR pathway, AMPK/mTOR pathway and GPX4 related pathway, which is very helpful for the combination of inhibitors. The combined targeting of mTOR and ferroptosis provides a potential intervention means for tumor cells. Thus, further studies on the molecular mechanisms associated with drug targeting of mTOR and ferroptosis could provide new therapeutic insights in the anti-tumor field.

## Data Availability

All data included in this study are available upon request by contact with the corresponding author.

## References

[1] Murugan AK (2019). mTOR: role in cancer, metastasis and drug resistance. Semin Cancer Biol.

[2] Zoncu R, Efeyan A, Sabatini DM (2010). mTOR: from growth signal integration to cancer, diabetes and ageing. Nat Rev Mol Cell Biol.

[3] Hirschhorn T, Stockwell BR (2019). The development of the concept of ferroptosis. Free Radical Biol Med.

[4] Xie Y, Lei X, Zhao G, Guo R, Cui N (2023). mTOR in programmed cell death and its therapeutic implications. Cytokine Growth Factor Rev.

[5] Bhattarai Y, Si J, Pu M (2021). Role of gut microbiota in regulating gastrointestinal dysfunction and motor symptoms in a mouse model of Parkinson's disease. Gut Microbes.

[6] Hua H, Kong Q, Zhang H, Wang J, Luo T, Jiang Y (2019). Targeting mTOR for cancer therapy. J Hematol Oncol.

[7] Saxton RA, Sabatini DM (2017). mTOR signaling in growth, metabolism, and disease. Cell.

[8] Morita M, Gravel S-P, Hulea L (2015). mTOR coordinates protein synthesis, mitochondrial activity and proliferation. Cell Cycle.

[9] Mader S, Lee H, Pause A, Sonenberg N (1995). The translation initiation factor eIF-4E binds to a common motif shared by the translation factor eIF-4 gamma and the translational repressors 4E-binding proteins. Mol Cell Biol.

[10] Gingras AC, Raught B, Sonenberg N (2001). Regulation of translation initiation by FRAP/mTOR. Genes Dev.

[11] Inoki K, Zhu T, Guan KL (2003). TSC2 mediates cellular energy response to control cell growth and survival. Cell.

[12] Gwinn DM, Shackelford DB, Egan DF (2008). AMPK phosphorylation of raptor mediates a metabolic checkpoint. Mol Cell.

[13] Ferrandiz-Pulido C, Masferrer E, Toll A (2013). mTOR signaling pathway in penile squamous cell carcinoma: pmTOR and peIF4E over expression correlate with aggressive tumor behavior. J Urol.

[14] Dworakowska D, Grossman AB (2009). The pathophysiology of pituitary adenomas. Best Pract Res Clin Endocrinol Metab.

[15] Nepstad I, Hatfield KJ, Grønningsæter IS, Reikvam H (2020). The PI3K-Akt-mTOR signaling pathway in human acute myeloid leukemia (AML) cells. Int J of Mol Sci.

[16] Liu GY, Sabatini DM (2020). mTOR at the nexus of nutrition, growth, ageing and disease. Nat Rev Mol Cell Biol.

[17] Peng F, Chang W, Sun Q (2020). HGF alleviates septic endothelial injury by inhibiting pyroptosis via the mTOR signalling pathway. Respir Res.

[18] Tong XP, Chen Y, Zhang SY (2014). Key autophagic targets and relevant small-molecule compounds in cancer therapy. Cell Prolif.

[19] Guan P, Wang N (2014). Mammalian target of rapamycin coordinates iron metabolism with iron-sulfur cluster assembly enzyme and tristetraprolin. Nutrition.

[20] Liu J, Zhang C, Wang J, Hu W, Feng Z (2020). The regulation of ferroptosis by tumor suppressor p53 and its pathway. Int J of Mol Sci.

[21] Gao W, Wang X, Zhou Y, Wang X, Yu Y (2022). Autophagy, ferroptosis, pyroptosis, and necroptosis in tumor immunotherapy. Signal Transduct and Target Ther.

[22] Huang Y, Wang S, Ke A, Guo K (2023). Ferroptosis and its interaction with tumor immune microenvironment in liver cancer. Biochim Biophys Acta.

[23] Gao M, Monian P, Quadri N, Ramasamy R, Jiang X (2015). Glutaminolysis and transferrin regulate ferroptosis. Mol Cell.

[24] Gkouvatsos K, Papanikolaou G, Pantopoulos K (2012). Regulation of iron transport and the role of transferrin. Biochim et Biophys Acta (BBA)— Gen Subj.

[25] Latunde-Dada GO (2017). Ferroptosis: role of lipid peroxidation, iron and ferritinophagy. Biochim Biophys Acta Gen Subj.

[26] Bresgen N, Eckl P (2015). Oxidative stress and the homeodynamics of iron metabolism. Biomolecules.

[27] Muckenthaler MU, Galy B, Hentze MW (2008). Systemic iron homeostasis and the iron-responsive element/iron-regulatory protein (IRE/IRP) regulatory network. Annu Rev Nutr.

[28] Miyake K, Nagai Y, Akashi S, Nagafuku M, Ogata M, Kosugi A (2002). Essential role of MD-2 in B-cell responses to lipopolysaccharide and Toll-like receptor 4 distribution. J Endotoxin Res.

[29] Liu Y, Liu X, Wang H, Ding P, Wang C (2022). Agrimonolide inhibits cancer progression and induces ferroptosis and apoptosis by targeting SCD1 in ovarian cancer cells. Phytomed : Int J of Phytother and Phytopharm.

[30] Cai S, Ding Z, Liu X, Zeng J (2023). Trabectedin induces ferroptosis via regulation of HIF-1α/IRP1/TFR1 and Keap1/Nrf2/GPX4 axis in non-small cell lung cancer cells. Chem Biol Interact.

[31] Yang Wan S, SriRamaratnam R, Welsch Matthew E (2014). Regulation of ferroptotic cancer cell death by gpX4. Cell.

[32] Hideyo Sato MT, Tetsuro Ishii, Bannai AS. Cloning and Expression of.pdf. J Biol Chem. 1999. 10.1074/jbc.274.17.11455 10206947

[33] Ursini F, Maiorino M (2020). Lipid peroxidation and ferroptosis: the role of GSH and GPx4. Free Radical Biol Med.

[34] Jia M, Qiu H, Lin L, Zhang S, Li D, Jin D (2022). Inhibition of PI3K/AKT/mTOR signalling pathway activates autophagy and suppresses peritoneal fibrosis in the process of peritoneal dialysis. Front Physiol.

[35] Fruman DA, Chiu H, Hopkins BD, Bagrodia S, Cantley LC, Abraham RT (2017). The PI3K pathway in human disease. Cell.

[36] Kim E, Kang JG, Kang MJ (2020). O-GlcNAcylation on LATS2 disrupts the hippo pathway by inhibiting its activity. Proc Natl Acad Sci U S A.

[37] Hassanein EHM, Abd El-Ghafar OAM, Ahmed MA (2020). Edaravone and acetovanillone upregulate Nrf2 and PI3K/Akt/mTOR signaling and prevent cyclophosphamide cardiotoxicity in rats. Drug Des Devel Ther.

[38] Horton JD, Goldstein JL, Brown MS (2002). SREBPs: activators of the complete program of cholesterol and fatty acid synthesis in the liver. J Clin Investig.

[39] Mahnke K, Becher E, Ricciardi-Castagnoli P, Luger TA, Schwarz T, Grabbe S (1997). CD14 is expressed by subsets of murine dendritic cells and upregulated by lipopolysaccharide. Adv Exp Med Biol.

[40] Zhao Y, Li M, Yao X (2020). HCAR1/MCT1 regulates tumor ferroptosis through the lactate-mediated ampk-scd1 activity and its therapeutic implications. Cell Rep.

[41] Magtanong L, Ko PJ, To M (2019). Exogenous monounsaturated fatty acids promote a ferroptosis-resistant cell state. Cell Chem Biol.

[42] Cheng Q, Chen M, Liu M (2022). Semaphorin 5A suppresses ferroptosis through activation of PI3K-AKT-mTOR signaling in rheumatoid arthritis. Cell Death Dis.

[43] Chen JN, Li T, Cheng L (2020). Synthesis and in vitro anti-bladder cancer activity evaluation of quinazolinyl-arylurea derivatives. Eur J Med Chem.

[44] Zhou B, Liu J, Kang R, Klionsky DJ, Kroemer G, Tang D (2020). Ferroptosis is a type of autophagy-dependent cell death. Semin Cancer Biol.

[45] Liu J, Kuang F, Kroemer G, Klionsky DJ, Kang R, Tang D (2020). Autophagy-dependent ferroptosis: machinery and regulation. Cell Chem Biol.

[46] Wei S, Qiu T, Yao X (2020). Arsenic induces pancreatic dysfunction and ferroptosis via mitochondrial ROS-autophagy-lysosomal pathway. J of Hazard Mater.

[47] Lin M, Hua R, Ma J (2021). Bisphenol A promotes autophagy in ovarian granulosa cells by inducing AMPK/mTOR/ULK1 signalling pathway. Environ Int.

[48] Du J, Wang T, Li Y (2019). DHA inhibits proliferation and induces ferroptosis of leukemia cells through autophagy dependent degradation of ferritin. Free Radical Biol Med.

[49] Zachari M, Ganley IG (2017). The mammalian ULK1 complex and autophagy initiation. Essays Biochem.

[50] Kim J, Kundu M, Viollet B, Guan KL (2011). AMPK and mTOR regulate autophagy through direct phosphorylation of Ulk1. Nat Cell Biol.

[51] Guo H, Ouyang Y, Yin H (2022). Induction of autophagy via the ROS-dependent AMPK-mTOR pathway protects copper-induced spermatogenesis disorder. Redox Biol.

[52] Bosch-Panadero E, Mas S, Civantos E (2018). Bisphenol A is an exogenous toxin that promotes mitochondrial injury and death in tubular cells. Environ Toxicol.

[53] Bao L, Zhao C, Feng L (2022). Ferritinophagy is involved in Bisphenol A-induced ferroptosis of renal tubular epithelial cells through the activation of the AMPK-mTOR-ULK1 pathway. Food Chem Toxicol.

[54] Kozma SC, Thomas G (1994). p70s6k/p85s6k: mechanism of activation and role in mitogenesis. Semin Cancer Biol.

[55] Akar U, Ozpolat B, Mehta K (2010). Targeting p70S6K prevented lung metastasis in a breast cancer xenograft model. Mol Cancer Ther.

[56] Zhang L, Liu W, Liu F (2020). IMCA induces ferroptosis mediated by slc7a11 through the ampk/mtor pathway in colorectal cancer. Oxid Med Cell Longev.

[57] Bell Eric L, Guarente L (2011). The SirT3 divining rod points to oxidative stress. Mol Cell.

[58] Singh CK, Chhabra G, Ndiaye MA, Garcia-Peterson LM, Mack NJ, Ahmad N (2018). The role of sirtuins in antioxidant and redox signaling. Antioxid Redox Signal.

[59] Han D, Jiang L, Gu X (2020). SIRT3 deficiency is resistant to autophagy-dependent ferroptosis by inhibiting the AMPK/mTOR pathway and promoting GPX4 levels. J Cell Physiol.

[60] Chen Y, Li N, Wang H (2020). Amentoflavone suppresses cell proliferation and induces cell death through triggering autophagy-dependent ferroptosis in human glioma. Life Sci.

[61] Sun Q, Zhen P, Li D, Liu X, Ding X, Liu H (2022). Amentoflavone promotes ferroptosis by regulating reactive oxygen species (ROS) /5'AMP-activated protein kinase (AMPK)/mammalian target of rapamycin (mTOR) to inhibit the malignant progression of endometrial carcinoma cells. Bioengineered.

[62] Dodson M, Castro-Portuguez R, Zhang DD (2019). NRF2 plays a critical role in mitigating lipid peroxidation and ferroptosis. Redox Biol.

[63] Hsieh CH, Hsieh HC, Shih FS (2021). An innovative NRF2 nano-modulator induces lung cancer ferroptosis and elicits an immunostimulatory tumor microenvironment. Theranostics.

[64] Tong L, Hao H, Zhang Z (2021). Milk-derived extracellular vesicles alleviate ulcerative colitis by regulating the gut immunity and reshaping the gut microbiota. Theranostics.

[65] Wu JC, Wang FZ, Tsai ML (2015). Se-Allylselenocysteine induces autophagy by modulating the AMPK/mTOR signaling pathway and epigenetic regulation of PCDH17 in human colorectal adenocarcinoma cells. Mol Nutr Food Res.

[66] Liu Y, Wang Y, Liu J, Kang R, Tang D (2021). Interplay between MTOR and GPX4 signaling modulates autophagy-dependent ferroptotic cancer cell death. Cancer Gene Ther.

[67] Zhang Y, Swanda RV, Nie L (2021). mTORC1 couples cyst(e)ine availability with GPX4 protein synthesis and ferroptosis regulation. Nat Commun.

[68] Thoreen CC, Kang SA, Chang JW (2009). An ATP-competitive mammalian target of rapamycin inhibitor reveals rapamycin-resistant functions of mTORC1. J Biol Chem.

[69] Ni J, Chen K, Zhang J, Zhang X (2021). Inhibition of GPX4 or mTOR overcomes resistance to Lapatinib via promoting ferroptosis in NSCLC cells. Biochem Biophys Res Commun.

[70] Kang R, Zhu S, Zeh HJ, Klionsky DJ, Tang D (2018). BECN1 is a new driver of ferroptosis. Autophagy.

[71] Li H, You L, Xie J, Pan H, Han W (2017). The roles of subcellularly located EGFR in autophagy. Cell Signal.

[72] Li H-W, Liu M-B, Jiang X, Song T, Feng S-X, Jing-ya Wu, Peng-fei D (2022). GALNT14 regulates ferroptosis and apoptosis of ovarian cancer through the EGFRmTOR pathway. Future Oncol.

[73] Wu X, Sheng H, Zhao L (2022). Co-loaded lapatinib/PAB by ferritin nanoparticles eliminated ECM-detached cluster cells via modulating EGFR in triple-negative breast cancer. Cell Death Dis.

[74] Napolitano G, Ballabio A (2016). TFEB at a glance. J Cell Sci.

[75] Li L, Sun S, Tan L (2019). Polystyrene nanoparticles reduced ROS and inhibited ferroptosis by triggering lysosome stress and tfeb nucleus translocation in a size-dependent manner. Nano Lett.

[76] Liu Y, Gu W (2022). The complexity of p53-mediated metabolic regulation in tumor suppression. Semin Cancer Biol.

[77] Shi L, Huang C, Luo Q (2020). Clioquinol improves motor and non-motor deficits in MPTP-induced. Aging.

[78] Yahagi N, Shimano H, Matsuzaka T (2003). p53 Activation in adipocytes of obese mice. J Biol Chem.

[79] Jiang L, Kon N, Li T (2015). Ferroptosis as a p53-mediated activity during tumour suppression. Nature.

[80] Ai Z, Lu Y, Qiu S, Fan Z (2016). Overcoming cisplatin resistance of ovarian cancer cells by targeting HIF-1-regulated cancer metabolism. Cancer Lett.

[81] Ichimura Y, Waguri S, Sou YS (2013). Phosphorylation of p62 activates the Keap1-Nrf2 pathway during selective autophagy. Mol Cell.

[82] Rawla P, Sunkara T, Barsouk A (2019). Epidemiology of colorectal cancer: incidence, mortality, survival, and risk factors. Prz Gastroenterol.

[83] Hinman A, Holst CR, Latham JC (2018). Vitamin E hydroquinone is an endogenous regulator of ferroptosis via redox control of 15-lipoxygenase. PLoS ONE.

[84] Chen H, Qi Q, Wu N (2022). Aspirin promotes RSL3-induced ferroptosis by suppressing mTOR/SREBP-1/SCD1-mediated lipogenesis in PIK3CA-mutatnt colorectal cancer. Redox Biol.

[85] Sung H, Ferlay J, Siegel RL (2021). Global cancer statistics 2020: GLOBOCAN estimates of incidence and mortality worldwide for 36 cancers in 185 countries. CA Cancer J Clin.

[86] Badgley MA, Kremer DM, Maurer HC (2020). Cysteine depletion induces pancreatic tumor ferroptosis in mice. Science.

[87] Cramer SL, Saha A, Liu J (2017). Systemic depletion of L-cyst(e)ine with cyst(e)inase increases reactive oxygen species and suppresses tumor growth. Nat Med.

[88] Egle Zalyte aJC. Proteomics Centre IoB, Vilnius University Life Sciences Centre. et al. Starvation mediates pancreatic cancer cell sensitivity to ferroptosis. Int J Mol Med. 2022. 10.3892/ijmm.2022.5140 PMC9106375 35514314

[89] Briganti A, Gandaglia G, Scuderi S (2020). Surgical safety of radical cystectomy and pelvic lymph node dissection following neoadjuvant pembrolizumab in patients with bladder cancer: prospective assessment of perioperative outcomes from the PURE-01 trial. Eur Urol.

[90] Sun Y, Berleth N, Wu W (2021). Fin56-induced ferroptosis is supported by autophagy-mediated GPX4 degradation and functions synergistically with mTOR inhibition to kill bladder cancer cells. Cell Death Dis.

[91] Kong N, Chen X, Feng J (2021). Baicalin induces ferroptosis in bladder cancer cells by downregulating FTH1. Acta Pharm Sin B.

[92] Hao J, Zhang W, Huang Z (2022). Bupivacaine modulates the apoptosis and ferroptosis in bladder cancer via phosphatidylinositol 3-kinase (PI3K)/AKT pathway. Bioengineered.

[93] Britt KL, Cuzick J, Phillips KA (2020). Key steps for effective breast cancer prevention. Nat Rev Cancer.

[94] Kwakye AK, Kampo S, Lv J (2020). Levobupivacaine inhibits proliferation and promotes apoptosis of breast cancer cells by suppressing the PI3K/Akt/mTOR signalling pathway. BMC Res Notes.

[95] Ma S, Henson ES, Chen Y, Gibson SB (2016). Ferroptosis is induced following siramesine and lapatinib treatment of breast cancer cells. Cell Death Dis.

[96] Pareja F, Reis-Filho JS (2018). Triple-negative breast cancers—a panoply of cancer types. Nat Rev Clin Oncol.

[97] Jaggupilli A, Ly S, Nguyen K (2022). Metabolic stress induces GD2(+) cancer stem cell-like phenotype in triple-negative breast cancer. Br J Cancer.

[98] Yi J, Zhu J, Wu J, Thompson CB, Jiang X (2020). Oncogenic activation of PI3K-AKT-mTOR signaling suppresses ferroptosis via SREBP-mediated lipogenesis. Proc Natl Acad Sci U S A.

[99] Ghoochani A, Hsu EC, Aslan M (2021). Ferroptosis inducers are a novel therapeutic approach for advanced prostate cancer. Cancer Res.

[100] Yangyun W, Guowei S, Shufen S, Jie Y, Rui Y, Yu R (2022). Everolimus accelerates Erastin and RSL3-induced ferroptosis in renal cell carcinoma. Gene.

[101] Wang J-Z, Zhu H, You P (2022). Upregulated YB-1 protein promotes glioblastoma growth through a YB-1/CCT4/mLST8/mTOR pathway. J of Clin Investig.

[102] Brown EJ, Albers MW, Shin TB (1994). A mammalian protein targeted by G1-arresting rapamycin-receptor complex. Nature.

[103] Hua H, Kong Q, Zhang H, Wang J, Luo T, Jiang Y (2019). Targeting mTOR for cancer therapy. J Hematol Oncol.

[104] Harvey RF, Pöyry TAA, Stoneley M, Willis AE (2019). Signaling from mTOR to eIF2α mediates cell migration in response to the chemotherapeutic doxorubicin. Sci Signal.

[105] Conlon M, Poltorack CD, Forcina GC (2021). A compendium of kinetic modulatory profiles identifies ferroptosis regulators. Nat Chem Biol.

[106] Cheung EC, Vousden KH (2022). The role of ROS in tumour development and progression. Nat Rev Cancer.

[107] Sarmiento-Salinas FL, Delgado-Magallón A, Montes-Alvarado JB (2019). Breast cancer subtypes present a differential production of reactive oxygen species (ROS) and susceptibility to antioxidant treatment. Front Oncol.

[108] Wu X, Xu Y, Liang Q (2022). Recent Advances in Dual PI3K/mTOR Inhibitors for Tumour Treatment. Front Pharmacol.

[109] Li M, Yang N, Hao L (2022). Melatonin inhibits the ferroptosis pathway in rat bone marrow mesenchymal stem cells by activating the pi3k/akt/mtor signaling axis to attenuate steroid-induced osteoporosis. Oxid Med Cell Longev.

[110] Lan D, Yao C, Li X (2022). Tocopherol attenuates the oxidative stress of BMSCs by inhibiting ferroptosis through the PI3k/AKT/mTOR pathway. Front Bioeng Biotechnol.

[111] Wang M, Cheng H, Wu H (2022). Gambogenic acid antagonizes the expression and effects of long non-coding RNA NEAT1 and triggers autophagy and ferroptosis in melanoma. Biomed Pharmacother.

